# Contextos de vulnerabilidade de adolescentes que (con)vivem com HIV: uma revisao integrativa*

**DOI:** 10.15649/cuidarte.2803

**Published:** 2023-09-08

**Authors:** Gabriel Pavinati, Lucas Vinícius de Lima, Marcelle Paiano, André Estevam Jaques, Gabriela Tavares Magnabosco

**Affiliations:** 1 Universidade Estadual de Maringá, Maringá, Brasil. Email: gabrielpavinati00@gmail.com Universidade Estadual de Maringá Universidade Estadual de Maringá Maringá Brazil gabrielpavinati00@gmail.com; 2 Universidade Estadual de Maringá, Maringá, Brasil. Email: lvl.vinicius@gmail.com Universidade Estadual de Maringá Universidade Estadual de Maringá Maringá Brazil lvl.vinicius@gmail.com; 3 Universidade Estadual de Maringá, Maringá, Brasil. Email: mpaiano@uem.br Universidade Estadual de Maringá Universidade Estadual de Maringá Maringá Brazil mpaiano@uem.br; 4 Universidade Estadual de Maringá, Maringá, Brasil. Email: aejaques@uem.br Universidade Estadual de Maringá Universidade Estadual de Maringá Maringá Brazil aejaques@uem.br; 5 Universidade Estadual de Maringá, Maringá, Brasil. Email: gtmagnabosco@uem.br Universidade Estadual de Maringá Universidade Estadual de Maringá Maringá Brazil gtmagnabosco@uem.br

**Keywords:** HIV, Adolescente, Saúde do Adolescente, Vulnerabilidade em Saúde, Revisao, HIV, Adolescent, Adolescent Health, Health Vulnerability, Review, VIH, Adolescente, Salud del Adolescente, Vulnerabilidad em Salud, Revisión

## Abstract

**Introdujo:**

Nos adolescentes, as vulnerabilidades decorrentes da infecto pelo HIV atrelam-se as singularidades biopsicossociais da fase, tornando-os um grupo prioritário para as estratégias de saúde.

**Objetivo:**

Analisar o estado da arte acerca das situacóes de vulnerabilidade de adolescentes que (con)vivem com HIV.

**Método:**

Revisao integrativa da literatura realizada em oito bibliotecas/bases de dados para responder a questao norteadora. Foram seguidas as recomendacóes padronizadas para revisao, os achados foram categorizados e discutidos de acordo com referencial da vulnerabilidade.

**Resultado:**

Foram identificadas 7.517 publicacóes, das quais 11 foram incluídas. Evidenciaram-se situacóes diversas de vulnerabilidade individuais, sociais e programáticas experienciadas por jovens com HIV, a saber: omissao do diagnóstico, estigma, discriminacao, baixa adesao a terapia antirretroviral, sofrimento emocional, entre outras.

**Discussao:**

Adolescentes que vivem com HIV sao suscetíveis a situacóes que os expóem a riscos reais e/ou potenciais. Nesse sentido, é imperioso qualificar os servicos e as acóes de saúde, em uma lógica de oferta universal e integral, livre de julgamentos baseados em crencas pessoais.

**Conclusao:**

Adolescentes que (con)vivem com HIV estao inseridos em contextos de vulnerabilidade dinámicos, subjetivos e complexos, cerceados por aspectos individuais, sociais e programáticos que influenciam negativamente o exercício de sua adolescencia, de sua saúde e de suas relacóes.

## Abstract (en)

**Introduction:**

In adolescents, the vulnerabilities resulting from HIV infection are linked to the biopsychosocial singularities of the phase, making them a priority group for health strategies.

**Objective:**

To analyze the state of the art regarding situations of vulnerability of adolescents who (co)live with HIV.

**Method:**

Integrative literature review conducted in eight libraries/databases to answer the guiding question. The standardized recommendations for review were followed, the findings were categorized and discussed according to the vulnerability framework.

**Result:**

7,517 publications were identified, of which 11 were included. Different situations of individual, social and programmatic vulnerability experienced by young people with HIV were evidenced, namely: omission of diagnosis, stigma, discrimination, low adherence to antiretroviral therapy, emotional distress, among others.

**Discussion:**

Adolescents living with HIV are susceptible to situations that expose them to real and/or potential risks. In this sense, it is imperative to qualify health services and actions, in a logic of universal and integral offer, free of judgments based on personal beliefs.

**Conclusion:**

Adolescents who (co)live with HIV are inserted in dynamic, subjective, and complex contexts of vulnerability, constrained by individual, social and programmatic aspects that negatively influence their adolescence, their health, and their relationships.

## Resumen

**Introducción:**

En los adolescentes, las vulnerabilidades derivadas de la infección por el VIH están ligadas a las singularidades biopsicosociales de la etapa, convirtiéndolos en un grupo prioritario para las estrategias de salud.

**Objetivo:**

Analizar el estado del arte sobre las situaciones de vulnerabilidad de los adolescentes que (co)viven con el VIH.

**Método:**

Revisión integrativa de la literatura realizada en ocho bibliotecas/bases de datos para responder a la pregunta guía. Se siguieron las recomendaciones estandarizadas para la revisión, los hallazgos se categorizaron y discutieron de acuerdo con el marco de vulnerabilidad.

**Resultado:**

se identificaron 7.517 publicaciones, de las cuales se incluyeron 11. Se evidenciaron diferentes situaciones de vulnerabilidad individual, social y programática que viven los jóvenes con VIH, a saber: omisión del diagnóstico, estigma, discriminación, baja adherencia a la terapia antirretroviral, angustia emocional, entre otras.

**Discusión:**

Los adolescentes que viven con VIH son susceptibles a situaciones que los exponen a riesgos reales y/o potenciales. En ese sentido, es imperativo calificar los servicios y acciones de salud, en una lógica de oferta universal e integral, libre de juicios basados en creencias personales.

**Conclusión:**

Los adolescentes que (co)viven con el VIH se insertan en contextos dinámicos, subjetivos y complejos de vulnerabilidad, constreñidos por aspectos individuales, sociales y programáticos que influyen negativamente en su adolescencia, su salud y sus relaciones.

## Introdujo

A adolescencia corresponde a fase da vida que se estende entre a infancia e a idade adulta, englobando aspectos decorrentes de alteragóes biológicas e transigóes sociais^1^^,^^2^. A ocorrencia paralela da puberdade precoce e do atraso na adaptagao dos papéis para a vida adulta tornam a conceituagao da adolescencia um enigma ao ponto que, sob o prisma social, entende-se que o período contempla indivíduos de 10 a 24 anos^1^.

Nesse momento ímpar da vida, existem constantes transformagóes e conflitos de ordem biológica, psicológica e social, reflexos da procura pela autonomia familiar, pelo protagonismo social e pela identidade individual^2^. Essa complexidade intrínseca a fase figura como uma vulnerabilidade para os adolescentes, sobretudo no que se refere ao exercício de saberes e práticas de saúde^3^.

Entende-se por vulnerabilidade as situagóes de exposigao de indivíduos a fatores e/ou eventos, sistematizados nas dimensóes individual, social e programática, que atuam de maneira interdependente para oportunizar a compreensao multidimensional dos influentes na saúde, tanto no ambito do adoecimento quanto nos estados social, mental, psicológico e físico decorrentes desse contexto^3^^,^^4^.

Dentre os cenários de vulnerabilidade entre os adolescentes, destaca-se a infecgao pelo vírus da imunodeficiencia humana (HIV). A iniciagao precoce da vida sexual e a adogao de comportamentos de risco sao aspectos que tornam o público mais suscetível a contrair a infecgao^5^. Somado a isso, tem- se a baixa percepgao de risco e a ausencia de atitudes protetoras, como consequencia do escasso conhecimento em torno do HIV^5^^,^^6^.

Estudo realizado na África do Sul identificou tendencia crescente do HIV entre adolescentes de 12 a 19 anos, com maior carga em meninas desempregadas e evadidas do ambiente escolar^7^. Esses achados sugerem que a vulnerabilidade deve ser compreendida em suas dimensóes de forma interrelacionada^3^^,^^4^, uma vez que fatores individuais, sociais e programáticos sao igualmente importantes na infecgao pelo HIV.

Por outro lado, é impreterível ressaltar que a complexidade das vulnerabilidades extrapola a exposigao ao virus. O fenómeno multifacetado do (con)viver com HIV abrange diferentes dominios que interferem na qualidade de vida das pessoas acometidas, refletindo em constructos que podem afetar negativamente o bem-estar e incitar a ocorrencia de fragilizagao das pessoas vivendo com HIV (PVHIV)^8^.

Nos adolescentes, as vulnerabilidades decorrentes da infecgao pelo HIV atrelam-se as singularidades biopsicossociais da fase, tornando-os um grupo prioritário para as estratégias de saúde. Portanto, empregar o aparato conceitual da vulnerabilidade na epidemia do HIV permite o reconhecimento do individuo e do seu contexto social e programático em uma lógica nao segmentada^9^.

Destarte, partindo do pressuposto de que os adolescentes correspondem a um grupo vulnerável frente as transigóes vivenciadas no adolescer e que o HIV tem o potencial de ocasionar vulnerabilidades em diversos aspectos da vida das pessoas infectadas, objetivou-se analisar, na literatura cientifica, o estado da arte acerca das situagóes de vulnerabilidade de adolescentes que (con)vivem com HIV.

## Materiais e Método

Tratou-se de uma revisáo integrativa da literatura, ancorada em seis etapas, a saber: (1) identificado do tema e pergunta de pesquisa; (2) definigáo dos critérios de inclusáo e exclusáo de estudos; (3) determinado das informagóes a serem extraídas dos estudos incluidos; (4) avaliagáo dos estudos incluídos; (5) análise dos resultados obtidos; e (6) síntese do conhecimento^10^.

Para construgáo da pergunta de pesquisa, utilizou-se os elementos do acrónimo Populado, Fenómeno de Interesse e Contexto (PICo)11. Para este estudo, postulou-se: P = adolescentes, I = situagóes de vulnerabilidade e Co = viver com HIV. Com base nessas definigóes, instituiu-se como questao norteadora: Quais sáo as situagóes de vulnerabilidade associadas a vivencia com HIV entre adolescentes?

Os critérios de inclusáo estabelecidos foram: estudos originais, nos idiomas portugués, ingles ou espanhol, que revelassem situagóes de vulnerabilidade de adolescentes decorrentes da vivencia com HIV. Para delimitagáo do público dos artigos elegíveis para esta revisáo, considerou-se como adolescente, enquanto construgáo social da fase, o indivíduo com idade entre 10 e 24 anos^1^ ^2^.

Os critérios de exclusáo definidos foram: publicagóes que náo estivessem disponíveis na íntegra, náo gratuitas, repetidas e provenientes da literatura cinzenta. Náo houve recorte temporal tendo em vista que as vulnerabilidades inerentes ao HIV compreendem um fenómeno singular e subjetivo, sendo necessário a análise e compreensáo da totalidade dos estudos disponíveis na literatura.

A busca aconteceu em junho de 2022 nas bases/bibliotecas: Web of Science (WOS); SCOPUS (Elsevier); Literatura Latino-Americana e do Caribe em Ciencias da Saúde (LILACS), Base de Dados em Enfermagem (BDENF) e Índice Bibliográfico Español en Ciencias de la Salud, via Biblioteca Virtual em Saúde (BVS); Scientific Medical Literature Analysis and Retrieval System (MEDLINE), via PubMed; Cummulative Index to Nursing and Allied Health Literature (CINAHL); e Biblioteca Virtual em Saúde do Adolescente (ADOLEC).

Os estudos foram acessados via Portal de Periódicos da Coordenagáo de Aperfeigoamento de Pessoal de Nível Superior (CAPES), por meio do acesso Comunidade Academica Federada (CAFe). Para sistematizagáo, utilizaram-se descritores das plataformas Descritores em Ciencias da Saúde (DeCS) e Medical Subject Headings (MeSH) (Tabela 1). Empregaram-se estratégias construídas por meio dos descritores e operadores booleanos “AND” e “OR” (Tabela 2).

**Tabela 1:** Descritores empregados para sistematizar a busca

PICo	Descritores controlados
P	Adolescente (Adolescent) e Saúde do Adolescente (Adolescent Health)
I	Vulnerabilidade em Saúde (Health Vulnerability),Vulnerabilidade Sexual (Sexual Vulnerability) e Vulnerabilidade Social (Social Vulnerability)
Co	HIV (HIV) e Síndrome da Imunodeficiência Adquirida (Acquired Immunodeficiency Syndrome)

**Tabela 2:** Estratégias de busca utilizadas de acordo com a base/biblioteca de dados

Base/biblioteca	Estratégia de busca
WOS	ALL=(Adolescentes OR "Saúde do adolescente" OR Adolescent OR “Adolescent Health”) AND ALL=(“Vulnerabilidade em Saúde” OR “Vulnerabilidade Sexual” OR “Vulnerabilidade Social” OR “Fatores de risco” OR “Health Vulnerability” OR “Sexual Vulnerability” OR “Social Vulnerability”) AND ALL=(HIV OR “Síndrome da Imunodeficiencia Adquirida” OR HIV OR “Acquired Immunodeficiency Syndrome”)
SCOPUS	TITLE-ABS-KEY(Adolescente OR "Saúde do adolescente" OR Adolescent OR “Adolescent Health”) AND TITLE-ABS-KEY(“Vulnerabilidade em Saúde” OR “Vulnerabilidade Sexual” OR “Vulnerabilidade Social” OR “Health Vulnerability” OR “Sexual Vulnerability” OR “Social Vulnerability”) AND TITLE-ABS-KEY(HIV OR “Síndrome da Imunodeficiencia Adquirida” OR HIV OR “Acquired Immunodeficiency Syndrome”)
LILACS BDENF IBECS	PALAVRAS(Adolescente) OR ("Saúde do adolescente") OR (Adolescent) OR (“Adolescent Health”) AND PALAVRAS(“Vulnerabilidade em Saúde”) OR (“Vulnerabilidade Sexual”) OR (“Vulnerabilidade Social”) OR (“Health Vulnerability”) OR (“Sexual Vulnerability”) OR (“Social Vulnerability”) OR AND PALAVRAS(HIV) OR (“Síndrome da Imunodeficiencia Adquirida”) OR (HIV) OR (“Acquired Immunodeficiency Syndrome”)
MEDLINE	Adolescent[Title] OR Adolescent Health[Title] AND Health Vulnerability[Title] OR Sexual Vulnerability[Title] OR Social Vulnerability[Title] AND HIV[Title] OR Acquired Immunodeficiency Syndrome[Title]
CINAHL	TX(Adolescente) OR ("Saúde do adolescente") OR (Jovem) OR (Adolescent) OR (“Adolescent Health”) AND TX(“Vulnerabilidade em Saúde”) OR (“Vulnerabilidade Sexual”) OR (“Vulnerabilidade Social”) OR (“Health Vulnerability”) OR (“Sexual Vulnerability”) OR (“Social Vulnerability”) OR (Repercussoes) OR (“Repercussoes sociais”) AND TX(HIV) OR (“Síndrome da Imunodeficiencia Adquirida”) OR (HIV) OR (“Acquired Immunodeficiency Syndrome”) OR (“Iníeccáo pelo HIV”)
ADOLEC	(Adolescentes) OR ("Saúde do adolescente") OR (Adolescent) OR (“Adolescent Health”) AND (“Vulnerabilidade em Saúde”) OR (“Vulnerabilidade Sexual”) OR (“Vulnerabilidade Social”) OR (“Health Vulnerability”) OR (“Sexual Vulnerability”) OR (“Social Vulnerability”) AND (HIV) OR (“Síndrome da Imunodeficiencia Adquirida”) OR (HIV) OR (“Acquired Immunodeficiency Syndrome”)

As recomendares do Preferred Reporting Items for Systematic Reviews and Meta-Analysis (PRISMA)^12^ ancoraram esta revisáo, gerando um fluxograma de selegáo contendo as fases de identificado, triagem, selegáo e inclusáo. Para além da busca nas bases/bibliotecas, procedeu-se a busca reversa nas referencias dos estudos incluidos, com vistas a assegurar maior captado de resultados. Todo o processo foi conduzido por dois pesquisadores de modo independente e as divergencias foram resolvidas por consenso.

Os estudos identificados nas bases/bibliotecas de dados e na busca reversa foram exportados para planilha eletronica. Para a organizado e apresentagáo de maneira resumida e objetiva, os dados foram organizados em: autor, ano, título, país de origem, tipo de estudo, abordagem, populado e vulnerabilidades (individual, social e programática). Para além disso, com vistas a facilitar a visualizado, foi desenvolvida uma nuvem de palavras das vulnerabilidades por meio da plataforma Mentimeter®.

Após a selegáo, procedeu-se a classificagáo por meio da Escala de Avaliagáo de Artigos com Metodologias Heterogéneas para Revisóes Integrativas (EAMH)^13^. A EAMH é composta por seis questóes dicotomicas (sim/náo) e sua pontuagáo varia de 0 a 6 pontos, sendo interpretada da seguinte forma: de 0 a 3 pontos = “artigo náo recomendado para análise”; 4 a 5 pontos = “artigo adequado para análise”; e 6 pontos = “artigo ideal para análise”^13^.

Para categorizagáo e discussáo dos achados, ancorou-se no referencial conceitual da vulnerabilidade proposto por Ayres^4^, que classifica os contextos em tres dimensóes: individual, social e programática. A primeira contempla os aspectos do modo de vida, dos saberes das pessoas sobre um assunto ea capacidade de empregá-los em práticas protetoras (conhecimentos, atitudes, comportamentos, relagóes afetivo-sexual, situagáo psicoemocional, situagáo física etc.)^4^.

A dimensáo social relaciona-se aos fatores contextuais da vida em sociedade e da rede de apoio social (normas sociais, estigma, discriminagáo, suporte social, cidadania, relagóes entre geragóes, dentre outros)^4^. Por fim, a dimensáo programática refere-se aos programas e servigos governamentais, seu efetivo funcionamento e qualidade (organizagáo do setor saúde, qualidade dos servigos, acesso aos servigos, preparo dos profissionais e afins)^4^.

O conjunto de dados deste estudo foi salvo no repositório público Mendeley Data®^14^. Cumpre mencionar que por se tratar de uma revisáo, cujas publicagóes incluídas encontram-se sob domínio público, náo foi necessária a apreciagáo pelo Comité de Ética em Pesquisa, conforme Resolugáo n° 466/2012 do Conselho Nacional de Saúde do Brasil. Ademais, ressalta-se que as ideias e conclusóes dos autores dos estudos incluídos foram mantidas, respeitadas e devidamente referenciadas, e os aspectos éticos foram observados.

## Resultados

**Figura 1:**
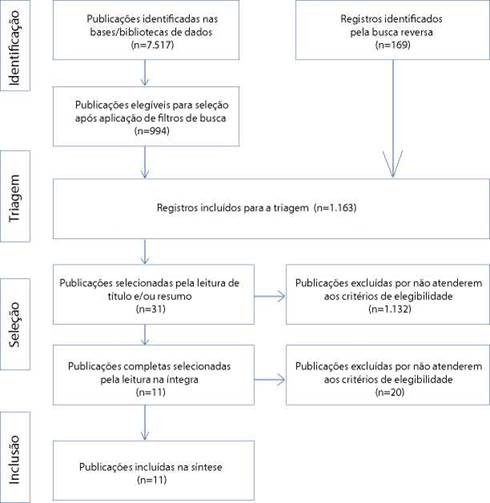
Fluxograma de seleáo dos estudos de acordo com as recomendares PRISMA

Foram identificadas 7.517 publicagóes. Após a aplicagáo dos filtros de busca, restaram 994 papers para a leitura de título e resumo, dos quais 22 foram selecionados, sendo incluídos, após leitura na íntegra, quatro estudos. Posteriormente, procedeu-se a busca reversa, pela qual retornou um total de 169 publicagóes, sendo nove selecionadas para leitura na íntegra e sete incluídas no estudo. Ao final, os estudos selecionados foram avaliados pela EAMH, com pontuagáo de 6 (n=9) e 5 (n=2), portanto, incluídos na revisáo (Figura 1).

Os estudos incluidos na revisao foram derivados do Brasil (n=5), do Malawi (n=3), de Gana (n=2) e da Uganda (n=1), e foram publicados entre 2009 e 2021. Houve maior frequéncia de estudos do tipo exploratório (n=4) e de abordagem qualitativa (n=8), desenvolvidos com adolescentes entre 11 e 24 anos. As vulnerabilidades relatadas compreenderam as dimensóes individual, social e programática (Tabela 3).

**Tabela 3:** Caracterizado dos estudos incluidos e vulnerabilidades identificadas

Autor e ano	Título e país	Tipo de estudo e abordagem	Populado	Vulnerabilidade		
				Individual	Social	Programática
Kaunda-Khangamwa et al, 2020^15^	Adolescents living with HIV, complex needs and resilience in Blantyre, Malawi Malawi	Estudo de caso Qualitativa	4 adolescentes entre 15 e 19 anos	Baixa adesáo a medicado, omis sao do diagnóstico ao parceiro e/ou família, comportamentos de risco e sofrimento emocional	Exclusáo familiar e social, estigma e discriminado	Dificuldade de acesso ao servido
Paula et al., 2013^16^	Cotidiano medicamentoso de adolescentes com HIV/AIDS Brasil	Transversal Quantitativa	23 adolescentes entre 13 e 19 anos	Omissáo do diagnóstico aos colegas e baixo conhecimento sobre a infecáo		
Doat et al., 2021^17^	Paradoxical experiences of Ghanaian adolescents with HIV: physiological challenges Gana	Exploratório Qualitativa	12 adolescentes entre 14 e 19 anos	Intensos sintomas físicos		Dificuldade de acesso ao servido, tratamento tardio
Silva et al., 2020^18^	Vulnerabilidade em saúde das jovens transexuais que vivem com HIV/ aids Brasil	Exploratório Qualitativa	6 adolescentes entre 18 e 24 anos	Baixo conhecimento sobre a infecáo	Exclusáo social, estigma e discriminado	
Enimil et al., 2015^19^	Quality of life among Ghanaian adolescents living with perinatally acquired HIV: a mixed methods study Gana	Estudo de métodos mistos	20 adolescentes entre 12 e 19 anos	Baixo conhecimento sobre a infecáo, omissáo do diagnóstico aos colegas e sofrimento emocional	Estigma e discriminado	
Kim et al., 2017^20^	High self-reported non-adherence to antiretroviral therapy amongst adolescents living with HIV in Malawi: barriers and associated factors Malawi	Transversal Quantitativa	519 adolescentes entre 12 e 18 anos	Baixa adesáo a medicado	Estigma e discriminado	Dificuldade de acesso ao servido
Ashaba et al., 2019^22^	Community beliefs, HIV stigma, and depression among adolescents living with HIV in rural Uganda Uganda	Exploratório Qualitativa	8 adolescentes entre 13 e 17 anos	Sofrimento emocional e físico, omissáo do diagnóstico para a família e ideaáo suicida	Exclusáo, - estigma, discriminado familiar/social e violencia familiar	
Kourrouski e Lima, 2009^23^	Adesáo ao tratamento: vivencias de adolescentes com HIV/aids Brasil	Descritivo Qualitativa	9 adolescentes entre 12 e 18 anos	Omissáo do diagnóstico aos colegas, náo aceitado do diagnóstico, baixa adesáo ao tratamento e ideaáo suicida	Estigma, -discriminado social e familiar	
Rodrigues et al., 2011^24^	Representares sociais de adolescentes e jovens vivendo com HIV acerca da adolescencia, sexualidade e aids Brasil	Exploratório Qualitativa	18 adolescentes entre 11 e 20 anos	Omissáo do diagnóstico, medo de transmitir a infecáo, baixa adesáo a medicado e baixo conhecimento sobre a infecáo	Estigma e - discriminado social	
Paula et al., 2011^25^	O (náo)dito da AIDS no cotidiano de transido da infancia para a adolescencia Brasil	Fenomenológico Qualitativa	11 adolescentes entre 11 e 14 anos	Omissáo do diagnóstico aos colegas e sofrimento emocional	Preconceito-	

**Figura 2:**
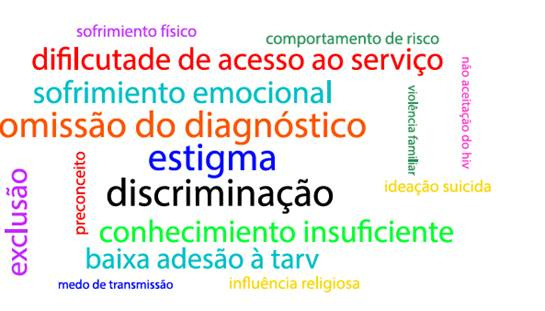
Nuvem de palavras das vulnerabilidades identificadas na revisao

Com relagáo as vulnerabilidades identificadas, observou-se situagóes diversas que sao experienciadas por jovens com HIV, sendo as mais frequentes: omissáo do diagnóstico (n=8), estigma (n=8), discriminagáo (n=8), baixa adesáo a terapia antirretroviral (TARV) (n=5), sofrimento emocional (n=5), entre outras. Os termos foram padronizados para a construgáo da Figura 2.

## Discussao

Os resultados desta revisao apontaram para as diversas situagóes de vulnerabilidade individual, social e programática vivenciadas por adolescentes com HIV. Sabe-se que o conceito de vulnerabilidade se aplica, comumente, a ideia de suscetibilidade a um agravo^26^, contudo, entende-se a necessidade de compreender os contextos para além do risco a doenga, compreendendo os aspectos do (con)viver com determinada condigao.

A análise das vulnerabilidades integra tres eixos correlacionados, abrangendo os aspectos da vida dos indivíduos, que os tornam mais suscetíveis a determinadas situagóes de risco^4^. Desse modo, busca-se interpretar e atribuir sentidos e significados aos fenómenos em estudo, com vistas a compreende-los em sua integralidade, de forma dinámica e multifacetada^4^.

Nesta revisao, essa compreensao busca alcangar maior articulagao das agóes e das estratégias de saúde ofertadas ao público adolescente, expandindo as intervengóes desenvolvidas para as tres esferas de vulnerabilidade e as influencias por elas exercidas, considerando o caráter complexo do objeto saúde-doenga e de sua causalidade^26^, representados, neste caso, pelo HIV.

A epidemia do HIV tem passado por constantes processos desde a sua descoberta. No contexto atual, evidencia-se a “juvenizagao” da epidemiologia da infecgao, com altos índices de morbimortalidade entre adolescentes em razao da parcela considerável de indivíduos que desconhecem seu status sorológico e das altas taxas de perda de seguimento do tratamento^27^.

Nessa lógica, compreende-se que os adolescentes que vivem com HIV estao suscetíveis a inúmeras situagóes que os expóem a um risco real e/ou potencial. Para essa compreensao, a ideia de vulnerabilidade deve se sobrepor ao conceito de risco, a medida que considera os contextos e relagóes sociais no processo de “vulnerabilizagao”, nao aceitando-o como estado natural^28^.

As PVHIV demandam agóes de saúde que transcendam os aspectos biológicos e clínicos da infecgao, englobando características sociais, individuais e culturais^29^. Essa especificidade, associada ao caráter de cronicidade do HIV, é permeada por debilidades programáticas, as quais refletem na oferta de assistencia fragmentada, centralizada e com enfoque biológico^29^.

Essa abordagem é incapaz de contemplar os diferentes contextos em torno da vida das PVHIV, uma vez que considera apenas elementos clínicos. Frente ao exposto, faz-se necessário apontar que a abordagem de manejo para a complexidade da infecgao pelo HIV deve pautar-se na intersetorialidade, mobilizando os atores sociais e políticos e efetivando os cuidados e as agóes ofertadas, sobretudo aos vulneráveis^29^.

Tal vulnerabilidade precisa ser compreendida enquanto uma estrutura conceitual, dinámica e interdependente, e nao que cristaliza a realidade^9^. Para tanto, é imperioso dar visibilidade a essas situagóes^28^. Nesse sentido, esta revisao destaca-se ao propor a identificagao e a sistematizagao dos aspectos individuais, sociais e programáticos que permeiam a vida dos adolescentes que vivem com HIV.

Na dimensao individual foram identificadas situagóes de omissao do diagnóstico ao parceiro, família e/ou colegas^15^^,^^16^^,^^19^^,^^21^^-^^25^, sofrimento emocional e/ou físico^15^^,^^17^^,^^19^^,^^21^^-^^25^, baixo conhecimento sobre a infecgao^16^^,^^18^^,^^19^^,^^24^, baixa adesao a TARV^15^^,^^20^^,^^23^^,^^24^^),^ ideagao suicida^22^^,^^23^, nao aceitagao do diagnóstico^23^, comportamentos de risco^15^ e medo de transmitir a infecgao^24^.

Destarte, percebe-se que essas vulnerabilidades individuais podem refletir em pontos cruciais na vida pessoal dos adolescentes, bem como em questóes de saúde em torno de seu (auto)cuidado. Todavia, esses aspectos nao devem ser considerados de maneira isolada, tendo em vista que, a luz conceitual da vulnerabilidade, as dimensóes atuam de maneira interrelacionada^4^.

Nessa perspectiva, enfatiza-se que os fatores evidenciados no contexto individual podem se relacionar ou, até mesmo, se originar das situagóes involucradas na dimensao social da vida das PVHIV, na qual observou-se a presenga de estigma^15^^,^^18^^-^^24^, discriminagao^15^^,^^18^^-^^24^, exclusao familiar e/ou social^15^^,^^18^^,^^21^^,^^22^^),^ preconceito^25^, violencia familiar^22^ e influencias religiosas^21^.

Sabe-se que o estigma em torno do HIV ocasiona atitudes de discriminagao, violencia e exclusao a nível estrutural, corroborando de diversas maneiras com as faces de vulnerabilidade individual, social e programática, de modo a produzir e reproduzir um universo de iniquidades sociais e de saúde^30^, sendo, portanto, resultados injustos e evitáveis no cenário da sociedade.

Para além das dimensóes individual e social, associa-se a programática, cujas vulnerabilidades relacionaram-se a dificuldade de acesso ao servigo^15^^,^^17^^,^^20^, ao diagnóstico e/ou tratamento tardio^17^, e a assistencia influenciada por questóes religiosas^21^. Assim, evidencia-se que a interlocugao entre os contextos da vulnerabilidade gera, de maneira interdependente, as situagóes vivenciadas por adolescentes com HIV.

O estigma e a discriminagao decorrentes do diagnóstico do HIV, historicamente impregnados na sociedade^31^, ampliam a precariedade da vida das PVHIV^28^. A persistencia dessa problemática tem ameagado e prejudicado as conquistas relacionadas a prevengao e ao cuidado dessas pessoas^32^. Esse cenário denota a necessidade de qualificar os servigos e as agóes de saúde, em uma lógica de oferta universal e integral, livre de julgamentos baseados em crengas pessoais.

Para tanto, aponta-se que o enfrentamento do fenómeno que o estigma do HIV representa deve compreender práticas estruturais, culturais e psicossociais, reconhecendo a origem social da estigmatizagao para que, assim, essa problemática possa ser desnaturalizada^31^. Assim, superar as atitudes balizadas pelo estigma parece ser primordial para a redugao das vulnerabilidades evidenciadas.

Nessa conjuntura, os profissionais da saúde envolvidos no cuidado as PVHIV desempenham papel de destaque^31^. No ámbito do Sistema Único de Saúde, o espago da Atengao Primária a Saúde (APS) figura como possibilidade de enfrentamento do status quo, uma vez que conhece as necessidades dos indivíduos adscritos e é capaz de desenvolver estratégias singularizadas de cuidado integral e de criagao de vínculo^32^.

A educagao permanente em saúde (EPS), enquanto processo de (re)construgao das práticas de cuidado a partir da realidade contextual, desponta como estratégia para a construgao de reflexóes que corroboram a construgao de novas configuragóes assistenciais e fomentam o cuidado qualificado^32^, tornando o profissional capaz de abordar a dimensao histórico-cultural, socioeconómica e política do HIV^31^.

Dessa forma, suscita-se a compreensao e o avango nas estratégias de promogao de uma cultura de nao discriminagao as pessoas que vivem com HIV^30^, sobretudo aquelas vivenciando a adolescencia, as quais, para além da carga de determinagao e exclusao social exercida pela infecgao, enfrentam as vulnerabilidades relacionadas aos fatores intrínsecos desse ciclo da vida.

Como limitagóes deste estudo, aponta-se o baixo número de artigos captados nas bases/bibliotecas de dados por meio das estratégias de busca utilizadas, bem como a restrigao lingüística empregada nos critérios de inclusao. Ademais, cumpre mencionar que os papers encontrados se referiram a realidade brasileira e de alguns países africanos, impedindo a identificagao das vulnerabilidades vivenciadas em países desenvolvidos.

## Conclusao

O mapeamento do estado da arte na literatura científica acerca do tema evidenciou que os adolescentes que (con)vivem com HIV estao inseridos em contextos de vulnerabilidade dinámicos, subjetivos e complexos, cerceados por aspectos individuais, sociais e programáticos que influenciam negativamente o exercício de sua adolescencia, de sua saúde e de suas relagóes.

Vislumbra-se, entao, um cenário que requer esforgos dos atores envolvidos, a nível individual, social e programático, no sentido de perceber as vulnerabilidades e superar as iniquidades existentes. Nesse sentido, faz-se necessário estruturar e/ou operacionalizar políticas públicas que compreendam os adolescentes com HIV em suas singularidades, além de promover a cultura de nao estigmatizagao.

Ademais, evidenciou-se interrelagao entre as dimensóes da vulnerabilidade, com repercussóes do HIV nas esferas pessoal, social e de saúde dos adolescentes. Assim, tendo em vista a complexidade do (con)viver com HIV nessa fase da vida, novos estudos sao necessários para melhor compreender o fenómeno e identificar as vulnerabilidades que assolam o público adolescente.

Finalmente, cumpre destacar que os estudos sugerem a importáncia da abordagem integral acerca das vulnerabilidades das PVHIV na formagao dos profissionais da saúde, bem como no processo de EPS, com intuito de promover a melhor compreensao desses contextos de vulnerabilidade, de modo a embasar e subsidiar o planejamento de cuidado holístico e intersetorial.

## Footnotes

1 ICo Descritores controlados P Adolescente (Adolescent) e Saúde do Adolescente (Adolescent Health)

2 Vulnerabilidade em Saúde (Health Vulnerability),Vulnerabilidade Sexual (Sexual Vulnerability) e Vulnerabilidade Social (Social Vulnerability) Co HIV (HIV) e Síndrome da Imunodeficiencia Adquirida (Acquired Immunodeficiency Syndrome)
